# Factors that influence locum practice in public and faith-based hospitals in Malawi

**DOI:** 10.4102/hsag.v29i0.2470

**Published:** 2024-05-31

**Authors:** Mabel B.N. Chinkhata, Masauko Msiska, Rodwell Gundo

**Affiliations:** 1 Postgraduate Department, School of Nursing, Kamuzu University of Health Sciences, Lilongwe, Malawi; 2 Biomedical Sciences Department, School of Life Sciences and Allied Health Professions, Kamuzu University of Health Sciences, Lilongwe, Malawi

**Keywords:** employment, health resources, health personnel staffing and scheduling, resource-limited settings, workload, Malawi

## Abstract

**Background:**

Locum practice is a non-standard form of employment used to address the shortage of healthcare workers in hospitals. In Malawi, the locum practice is used to improvre the availability of healthcare workers and promote continuity of care. Despite this, little attention has been paid to the effective and efficient use of locum practice.

**Aim:**

To explore the factors influencing locum practices in public and faith-based hospitals in Malawi.

**Setting:**

Six public and faith-based hospitals in Malawi.

**Methods:**

An exploratory descriptive design was used in the qualitative strand of this larger mixed-methods study. Data were collected through in-depth interviews with hospital managers (*n* = 15) and focus group discussions (FGD) with nurses, medical officers, and clinical officers (*n* = 29). All the interviews were audio-recorded and transcribed verbatim. Thematic analysis was used to analyse the data.

**Results:**

Locum practice was characterised by challenges related to healthcare workers working beyond the prescribed hours, a lack of orientation for locu staff, inconsistent locum rates, and delayed payments. The consequences of these challenges are compromised quality and safety of patient care.

**Conclusion:**

Locum practice is associated with numerous challenges in both public and faith-based hospitals in resource-limited settings. This could be attributed to the lack of regulation, supervision and monitoring of locum practice.

**Contribution:**

The findings suggest the need for the development and adherence to guidelines and policies for managing locum practice. Further studies could explore measures to retain permanent staff as a way of reducing the demand for locum staff.

## Introduction

The shortage of healthcare workers is a global problem that necessitates the use of several strategies, such as locums, to increase the staffing. Globally, there is a projected shortfall of 10 million health workers by 2030 especially in low- and lower-middle-income countries (Liu et al. 2017; World Health Organization [WHO] 2020). Locum healthcare workers are used in health facilities to fill the gaps created by permanent healthcare workers who are on annual, sick, or maternity leave, or just fill a vacant position (Ferguson & Walshe 2019, 2019; Grigoroglou et al. 2022). Locum practice is a non-standard form of employment (Tekeli-Yesil & Kiran 2020) that is used to cover gaps in the rota for nurses, midwives, clinical officers (also known as associates), and medical officers (Lougheed 2017). Locum healthcare workers are also referred to as locum tenens healthcare workers. According to DeCapua (2016), locum tenens is a Latin word that is translated as ‘one holding a place’ and refers to anyone who temporarily fulfils the duties of another (DeCapua 2016). Healthcare workers are recruited through agencies (externally) and sometimes directly (internally) by a facility that requires the service (Stringer et al. 2023). Locum healthcare workers may be hired on daily or weekly basis leading to challenges with continuity of care (Salloch et al. 2018). Unfortunately, in Malawi, there are no agencies for the recruitment of locum staff; therefore, hospitals recruit locum staff directly and mostly the same permanent staff are used for locum shifts.

Malawi, like many sub-Saharan African countries, experiences a shortage of human resources for health, which affects the health services. The population to health worker ratio of 28.5 health workers for every 10 000 people is below the WHO target of 44.5 health workers per 10 000 people (WHO 2020). According to the country’s Health Sector Strategic Plan II, there are 62 269 established positions for healthcare workers for public and faith-based facilities, of which 39 494 (63%) are clinical, nursing, allied services, education, and allied technical staff (Government of the Republic of Malawi 2017). However, only 52% of these positions are filled. In response to this shortage, the government recruited 5622 healthcare workers between 2020 and 2022 to mitigate the shortage (Government of the Republic of Malawi 2023). However, the numbers remained below the anticipated demand. Several studies have reported associations between staffing levels and patient outcomes. The WHO has reported a strong positive correlation between health workforce density and health outcomes (WHO 2020). For example, a study conducted in Finland indicated that a 1% increase in nurse staffing levels would reduce the mortality rate by 0.65% (Amiri & Solankallio-Vahteri 2019; Dall’Ora et al. 2022). Therefore, locum staff are essential for maintaining the continuity of healthcare services and helping organisations to maintain appropriate staffing levels (Ferguson & Walshe 2019). The locum practice also provides healthcare workers with staff flexibility.

Although the use of locum healthcare workers in hospitals ensures the availability of staff, continuity of services, and improved quality patient care, there are also challenges related to the use of locum staff. The practice is associated with compromised patient safety because of increased workload on permanent staff, increased costs, reduced team function, and suboptimal quality of services (Ferguson & Walshe 2019; Miseda et al. 2017; Stringer et al. 2023; Xue et al. 2014), among other challenges. The literature shows that challenges can be reduced by placing guidelines in place for the appointment and assessment of locum staff to safeguard the quality of care (British Medical Association 2018; Keelson & Dodor 2014). According to Keelson and Dodor (2014), a lack of guidelines leads to many challenges with locum practice. Therefore, this paper presents the qualitative findings of a larger study that aimed to develop guidelines for the management of locum practices in the country’s public and faith-based hospitals. This qualitative study explores the factors that influence locum practice in public and faith-based facilities.

## Research methods and design

### Study design

A qualitative descriptive design underpinned by the constructivist paradigm was used in the first phase of this study. A qualitative design is flexible and allows for an in-depth examination of a phenomenon (Deterding & Waters 2018). Furthermore, qualitative methods reveal differences in viewpoints between groups by generating a wide range of ideas and opinions on issues (Creswell & Clark 2017). The approach was considered appropriate because it allows for the exploration of a variety of topics, the generation of new knowledge, or answers to unclear research questions about which little is known (Hallingberg et al. 2018).

### Population

The participants included medical officers, clinical officers, nurses, midwives, and the hospital management team at six public and faith-based hospitals. In Malawi, the hospital management team comprises the hospital director or medical director, head of nursing and midwifery services, and hospital administrators. Clinical officers, also known as clinical associates, are trained to perform minor operations, caesarean sections, and comprehensive emergency obstetric care services in cases where specialists are not available (Couper et al. 2018; Gajewski et al. 2018, 2019).

### Inclusion and exclusion criteria

Qualified and licensed medical doctors, clinical officers, nurses, midwives, and members of the management team who had worked at a facility for more than 6 months were included in the study. The participants were included because they have in-depth knowledge of locum practice or worked as locums. However, interns, student medical doctors, student clinical officers, student nurses, and midwives were excluded from the study because they are not qualified or licensed by a regulatory body to practise independently.

### Study setting

The study was conducted at six health facilities, which included two central (tertiary) hospitals, two district hospitals, and two faith-based hospitals in Malawi. Faith-based hospitals, also known as Christian Health Association of Malawi (CHAM) hospitals, are private not-for-profit facilities. This is the second largest service provider in Malawi, which owns 26% of the health facilities, after the Ministry of Health, and some of these facilities are as big as district hospitals. One of the sampled hospitals is located in the country’s northern region, two in the central region, and three in the southern region. In Malawi, central hospitals are tertiary-level facilities that serve as referral centres for secondary-level hospitals and provide specialised services. District hospitals provide secondary services and serve several primary facilities. Faith-based facilities are private not-for-profit facilities that provide services in rural areas at a small fee (Government of the Republic of Malawi 2017).

### Sample and sampling technique

A pragmatic sample of 44 participants (managers, *n* = 15; nurses, midwives, medical officers, and clinical officers, *n* = 29) was selected using purposive sampling. All the participants had knowledge about locum practice and were able to provide in-depth information. The sampling provided for maximal variation to ensure a mix of health workers from various departments with different levels of experience and perspective of locum practice. The demographic profiles of the participants are presented in Table 1.

**TABLE 1:** Demographic characteristics of individual-expert interview and focus group discussion participants.

Variables	Category	In-depth interview participants (IDI) (*n* = 15)	Focus group discussion participants (*n* = 29)
Gender	Male	9	11
	Female	6	18
Age (years)	Mean	41	34
Marital status	Single	3	11
	Married	12	18
Professional cadre	Nursing	6	21
	Clinical	6	8
	Administration	3	-
Qualification	Certificate	-	2
	Diploma	2	14
	BSc	8	13
	MSc	5	-
Years of experience	Mean	10	6

BSc, bachelor of science degree; MSc, master of science degree.

### Data collection

Participants who agreed to participate in the study were given an information sheet and requested to provide written consent. Two research assistants collected the data in the six facilities between June and September 2019. A research assistant with experience in qualitative studies was recruited from a research institution; therefore, it was easier for him to collect data for this study. The other assistant took notes during the interviews and the focus group discussions (FGDs). Both were nurses and knowledgeable about locum practices. Data were gathered through in-depth interviews (IDI) with hospital managers (*n* = 15) who are key informants and through three FGDs, one from each type of facility (central, district, and faith-based). Focus group discussions were conducted with nurses, midwives, medical officers, and clinical officers (*n* = 29). The interviews were conducted within the hospital at a time and place convenient for the participants. An interview guide with unstructured questions was used. Each in-depth interview and FGD session lasted 30 min – 60 and 60 min – 90 respectively. The interviews were audio-recorded and transcribed verbatim.

### Data analysis

The transcribed data were analysed using thematic analysis (Braun & Clarke, 2006, 2019). The approach involved familiarising oneself with the collected data, generating initial codes, searching for themes, reviewing the themes, defining and naming themes, and producing a report. NVivo software version 12.0 was used to organise the data into small, manageable units.

### Trustworthiness

The trustworthiness of the research findings was achieved through the following strategies: credibility, dependability, confirmability, and transferability (Polit & Beck 2017). Credibility was achieved through prolonged engagement and triangulation. Method, person, and space triangulation were achieved using different methods of data collection, the inclusion of different healthcare workers from different hospitals, and prolonged engagement. A detailed account of the research process and study setting was provided to ensure transferability. Dependability was achieved through the creation of a database of proposal drafts (when, how, and why they changed), memos, field notes, anonymised filled questionnaires, and transcripts for potential audit trails and secondary analysis. Confirmability was achieved by including excerpts from the transcribed texts that connected data and the findings.

### Ethical considerations

Hospital managers provided permission to conduct the study at the six study hospitals (The College of Medicine Research Ethics Committee [COMREC], certificate number P.04/19/2648). Each participant provided written consent after going through the study information sheet and all ethical requirements for the study. Confidentiality and privacy were maintained by using pseudonyms during the interviews.

## Results

These results were based on the in-depth interviews with senior managers and focus group discussions with frontline workers such as nurses, midwives, medical doctors, and clinical officers. The demographic characteristics of the participants are shown in Table 1. The majority of the participants were older than 35 years. However, their ages ranged from 30 years to 54 years old. The sample reflects the fact that nurses and midwives comprise the majority of the healthcare workers in the health system. It suffices to say that most management team members are males than females with varying qualifications and work experience.

### Theme and subthemes

The dominant theme was compromised quality of care, which resulted from challenges in locum practices. The participants in all hospitals reported that locum practice is marred by numerous challenges that are related to healthcare workers *working beyond the prescribed hours, a lack of orientation for locum staff*, and *inconsistent locum rates and delayed payments* (as shown in Table 2). Consequently, these challenges compromise the quality of patient care.

**TABLE 2:** Theme and subthemes.

Theme	Subthemes
Locum practice deemed a challenge to patient care	Healthcare workers working beyond the prescribed hours
	Lack of orientation for locum staff
	Inconsistent locum rates and delayed payments

### Healthcare workers working beyond the prescribed hours

The participants revealed that locum healthcare workers in all hospitals work beyond the government-prescribed hours (40 h) per week. After working for the prescribed hours, it becomes imperative for the healthcare workers to continue with locum shifts to cover the gaps without proper rest. Therefore, the use of the same staff for normal and locum shifts deprives healthcare workers of rest and compromises their performance and quality of patient care, as reported by two participants from public hospitals:

‘We have over-utilised our people; our staff, instead of getting rest, they are forced to come and do locum which compromises their performance.’ (Participant 10, Male, 37 years)

‘You overwork and the care provided when one is tired, is compromised.’ (Participant 6, Female, 28 years)

In addition, a manager from a faith-based facility observed that healthcare workers, especially nurses and midwives who are not permanently employed at any facility, work more shifts on locum without rest because they want to impress their employers to be considered for permanent employment:

‘When they work on locum, they become dedicated to impress the supervisor so that they can be employed. As a result each time they are called for a shift they do not refuse, they get as many shifts allocated to them; sometimes they work day-night-day.’ (Participant 14, Female, 47 years)

In agreement, another manager at one of the central hospitals said that sometimes healthcare workers do not want to rest; they work continuously because they are motivated with the money for locum, as reported:

‘Some people when they see the money they are willing to work all their lungs out they do not want to go and rest, so although we want people to work again, we try our best to adhere to the set guidelines or policy.’ (Participant 4, Male, 40 years)

However, the clinical officers reported that they do not work continuously because their shifts do not allow them to do so. They work based on calls, which are already 24 h with 4 h of rest, and once the shift is done, they go and rest:

‘[*O*]ur calls are not continuous; when you work today, tomorrow you will rest.’ (Participant 18, Male, 31 years)

In contrast, a participant from one central hospital reported that in their facility, nurses did not work in continuous shifts. The nurses used to work continuous shifts called *Petroda*, to save on transport expenses, but the practice has stopped. Nurses are now encouraged to rest before a shift to be effective:

‘We used to have nurses working what they call Petroda; they work during the day and continue to work during the night. Working 24 hours continuously compromised the quality of care because you cannot work continuously and be effective.’ (Participant 10, Male, 37 years)

### Lack of orientation for locum staff

Participants reported that sometimes the hospitals use locum staff from other health facilities or departments. Healthcare workers from other facilities may not be familiar with the work environment; therefore, they may require orientation. However, none of the participants observed that locum staff had not received adequate orientation:

‘When we employ these locum people they do not undergo this one-week orientation, but we orient them for a few days, and it is not as intensive as the other ones.’ (Participant 9, Male, 42 years)

When the locum staff are not orientated to protocols and procedures, they do not perform according to the hospital’s standards, as reported by one manager:

‘When the locum person is not oriented to the hospital’s protocols they may perform some procedures differently and patients may lose trust in them. Sometimes the patient reports that the nurse that we had did not do this in the manner that you always do.’ (Participant 12, Male, 39 years)

In the absence of proper orientation, some cadres, especially medical officers and clinical officers of faith-based hospitals, developed a list of policies and protocols that locum staff must be aware of. For example, one manager said that they had a list of things that each doctor and clinical officer needed to know about the hospital and its policies. The list is given to every medical officer and clinical officer who works in the facility. A clinician from one of the faith-based facilities also echoed this:

‘We have got a list of what each doctor should know and how to carry themselves within the hospital. We give the list to every clinician that come to our hospital to make sure that they know about the policies we have before touching patients.’ (Participant 1, Female, 55 years)

### Inconsistent locum rates and delayed payments

These findings indicated that health workers are dissatisfied and disgruntled with the locum rates and delays in payments. Because of dissatisfaction, the care provided was reported to be of a low standard, and sometimes other activities were left undone. Some participants complained about low locum payment rates compared to the workload. The participants reported that most healthcare workers that do locums are not satisfied with the locum rate and this has led to some permanent staff not committing to the locum work:

‘The workers are not happy with the rates because the money is too little considering living expenses and the amount of work done.’ (Participant 21, Female, 40 years)

However, other participants observed that some faith-based facilities paid higher rates than public facilities to attract more staff. For example, a participant from one faith-based hospital said:

‘[*W*]hat we normally change are the rates; for us we raised the rates a little bit to make it attractive for our staff and other people.’ (Participant 2, Female, 54 years)

Although some facilities paid higher rates, others used the same stipulated rates:

‘We pay a government rate.’ (Participant 6, Female, 28 years)

Consequently, the staff is dissatisfied with locum rates, which causes them not to perform well:

‘A patient could have received more care; they could have been cared for even better if the staff were satisfied with what they get, they could have produced more.’ (Participant 13, Male, 26 years)

In addition, the participants, especially the frontline workers, reported that the locum payments were usually late, and this was reported in almost all facilities. They indicated that healthcare workers expected payment soon after their shifts. However, in some facilities, it takes 2 months to 4 months for healthcare workers to be paid, as reported by one of the nurses:

‘If you work this month, then you get your pay next month. Sometimes it takes 2–3 months even 4 months before you get paid.’ (Participant 3, Female, 24 years)

However, some managers do not agree with the reports that locum payments are delayed. For instance, a manager from a faith-based facility said that the facility was paying immediately:

‘We do not experience such instances where payments are delayed for more than a month. We rarely do that; as CHAM hospitals, we do not exceed a month. We pay immediately.’ (Participant 2, Female, 54 years)

Similarly, a manager from a government facility reported that they paid locum staff in their facilities at the end of the month. However, the manager admitted that there are delays in processing payments for those working for the first time because of the government payment systems:

‘Sometimes, it happens that you work for locum this month but you do not receive the money at the end of the month and you may receive it after a month or two. This is attributed to government payment system.’ (Participant 12, Male, 39 years)

## Discussion

This study aimed to explore the factors affecting locum practice in public and faith-based hospitals in Malawi. The findings revealed challenges associated with locum practice as follows: healthcare workers, especially nurses, worked for more than the government prescribed weekly hours, a lack of orientation of locum staff, inconsistent locum rates, and delays in payments. These challenges lead to dissatisfaction, demotivation, frustration, compromised performance, and a poor quality of patient care.

The study revealed that locum staff overworked to increase their financial gains. Financial gains influence the use of locum healthcare workers. Healthcare workers mentioned that they engaged in locum practices to increase their incomes. However, this resulted in the staff working more shifts in order to increase their chances of having more money. Similarly, in South Africa, 72.5% of the nurses said that more money was the reason for engaging in agency nursing (Rispel et al. 2014). Although this was related to excessive working hours, it also negatively affects patient care. In Malawi, permanent healthcare workers are expected to work 40 h per week. However, with the shortage of staff, the same healthcare workers may work additional shifts to cover the gaps in the rota within the same week, thus influencing the quality of care. The study revealed that hospitals use the same permanent staff for locums, resulting in locum healthcare workers working 32 h more per week. The finding points to the need for tighter management and regulation and improved supervision and monitoring of locum practices.

Hospitals use the same permanent staff to work as locums within the hospital. However, they may recruit staff to work at a workstation (department) different from their primary station. Under certain circumstances, staff from other hospitals may be recruited to the locum practice. In both scenarios, healthcare workers are unfamiliar with the work environment, so they require orientation and induction (Blumenthal et al. 2017). According to Blumenthal et al. (2017), locum tenens physicians struggle to deliver care effectively and efficiently in unfamiliar environments because of a lack of induction. The lack of familiarity with the institution and its protocols, which was compounded by the lack of orientation, was reported to be one of the challenges of locum practices that leads to poor quality of care (Bajorek & Guest 2019; Borek et al. 2022). Despite their qualifications and competencies, healthcare workers are less likely to perform well in unfamiliar settings or working environments. Bajorek and Guest (2019) revealed that managers spent little time on induction or orientation of agency nurses. Similarly, insufficient hospital induction has been reported in the UK, where the lack of such prerequisites deprives doctors of the information essential for their practice (Theodoulou, Reddy & Wong 2018).

The current study has revealed the need to orient staff members who are not working at their primary workstation according to the protocols and procedures of the department. Although some hospitals, especially faith-based hospitals, oriented their locum staff, this was reported to be inadequate. When induction is inadequate, the locum healthcare worker is more reliant on the other permanent staff available on the shift even for very simple activities. This can lead to inefficiencies and compromised patient safety if the ward or department is too busy (Stringer et al. 2023). Therefore, the orientation or induction to the protocols and procedures is critical for all external and internal staff to reduce potential risks to patient care (Borek et al. 2022). Borek et al. (2022) recommend hospitals to recruit and allocate locum staff at one local area and for regular long-term to reduce the challenge of unfamiliarity. Hospitals that engage locum staff need to plan for appropriate induction, allocate more time for familiarisation, and emphasise on adherence to guidelines, policies, and procedures.

Furthermore, concerns have been raised regarding inconsistent rates and delayed payments for locum practices. Inconsistent rates and delayed payments negatively influenced the engagement and recruitment of health care workers in locum work. Locum practice is perceived as a financial gain and key motivation for the locum tenens healthcare workers (Ferguson et al. 2021). Inconsistent rates and delayed payments demotivate providers leading to poor quality and safety of care (Ferguson et al. 2021). When staff are not given what is due to them in terms of salaries and locum allowances, they become frustrated, demotivated, and even have intentions to leave the health system (Chimwaza et al. 2014). Therefore, delays in locum payment lead to demotivation considering that their salaries are already low and some depend solely on the locum payment which have inconsistent rates. The study revealed that other hospitals were facing challenges in recruiting locum staff because of delays and the low locum rates. Therefore, it is necessary to ensure consistent and timely payments to retain and maintain the locum staff. Hospitals should include locum payments on the budget line to reduce delays. It is not uncommon for locum tenens to work without benefits, and without the ability to participate in other payment programmes. Contrary to these findings, locums in Malawi are paid per shift. Studies from other countries reported that locum healthcare workers are paid per hour, which is higher than the pay for permanent staff. For example, a study in United Kingdom (UK) revealed that hourly locum rates were reported to be higher than those of full-time employees, which entices locum staff to continue working in locum shifts (Theodoulou et al. 2018).

The reported financial problems have the potential to demotivate staff and lead to job dissatisfaction. In Malawi, the locums are paid from the Other Recurrent Transaction (ORT) budget, which is also used to purchase drugs, medical supplies, and other operations. This sometimes leads to challenges in the prioritisation of the budget line items, affecting less locum payments in some months. The health budgetary allocation is mostly below 15% of the national budget, as stipulated by the Abuja declaration (Biegon 2020). For example, in the 2020–2021 fiscal year, the government allocated 9.0% of the national cake to health (Unicef Malawi Health 2021). In addition, the health sector’s budget is largely (75%) dependent on foreign resources, leading to unstable and unpredictable health financing. Therefore, managers need to lobby for employment and the deployment of more permanent staff to reduce the vacancy rate and hire locum health workers.

## Conclusion and recommendations

Although locums are perceived as a source of additional staff, several factors negatively affect locum practice. The consequences of these challenges are compromised quality and safety of patient care. Hospitals need to plan for the difficulties of working in an unfamiliar environment. They should give appropriate induction to the locum staff, and allocate more time for familiarisation and set up systems, policies, and procedures to be followed. Hospital managers should be familiar and adhere to guidelines for the management of locums. The findings suggest the need for managers to lobby for resources for locum practice and explore measures to retain permanent staff as a way of reducing the demand for locum practice in resource-limited settings, such as Malawi.
